# ***In vitro*assembly of the bacterial actin protein MamK from ‘*Candidatus*Magnetobacterium casensis’ in the phylum*Nitrospirae***

**DOI:** 10.1007/s13238-016-0253-x

**Published:** 2016-03-09

**Authors:** Aihua Deng, Wei Lin, Nana Shi, Jie Wu, Zhaopeng Sun, Qinyun Sun, Hua Bai, Yongxin Pan, Tingyi Wen

**Affiliations:** CAS Key Laboratory of Pathogenic Microbiology and Immunology, Institute of Microbiology, Chinese Academy of Sciences, Beijing, 100101 China; Biogeomagnetism Group, Paleomagnetism and Geochronology Laboratory, Key Laboratory of Earth and Planetary Physics, Institute of Geology and Geophysics, Chinese Academy of Sciences, Beijing, 100029 China; University of Chinese Academy of Sciences, Beijing, 100049 China

**Keywords:** magnetotactic bacteria, *Nitrospirae*, bacterial actin, MamK, assembly mechanism

## Abstract

Magnetotactic bacteria (MTB), a group of phylogenetically diverse organisms that use their unique intracellular magnetosome organelles to swim along the Earth’s magnetic field, play important roles in the biogeochemical cycles of iron and sulfur. Previous studies have revealed that the bacterial actin protein MamK plays essential roles in the linear arrangement of magnetosomes in MTB cells belonging to the *Proteobacteria* phylum. However, the molecular mechanisms of multiple-magnetosome-chain arrangements in MTB remain largely unknown. Here, we report that the MamK filaments from the uncultivated ‘*Candidatus* Magnetobacterium casensis’ (Mcas) within the phylum *Nitrospirae* polymerized in the presence of ATP alone and were stable without obvious ATP hydrolysis-mediated disassembly. MamK in Mcas can convert NTP to NDP and NDP to NMP, showing the highest preference to ATP. Unlike its *Magnetospirillum* counterparts, which form a single magnetosome chain, or other bacterial actins such as MreB and ParM, the polymerized MamK from Mcas is independent of metal ions and nucleotides except for ATP, and is assembled into well-ordered filamentous bundles consisted of multiple filaments. Our results suggest a dynamically stable assembly of MamK from the uncultivated *Nitrospirae* MTB that synthesizes multiple magnetosome chains per cell. These findings further improve the current knowledge of biomineralization and organelle biogenesis in prokaryotic systems.

**Electronic supplementary material:**

The online version of this article (doi:10.1007/s13238-016-0253-x) contains supplementary material, which is available to authorized users.

## **INTRODUCTION**

**Figure 1:**
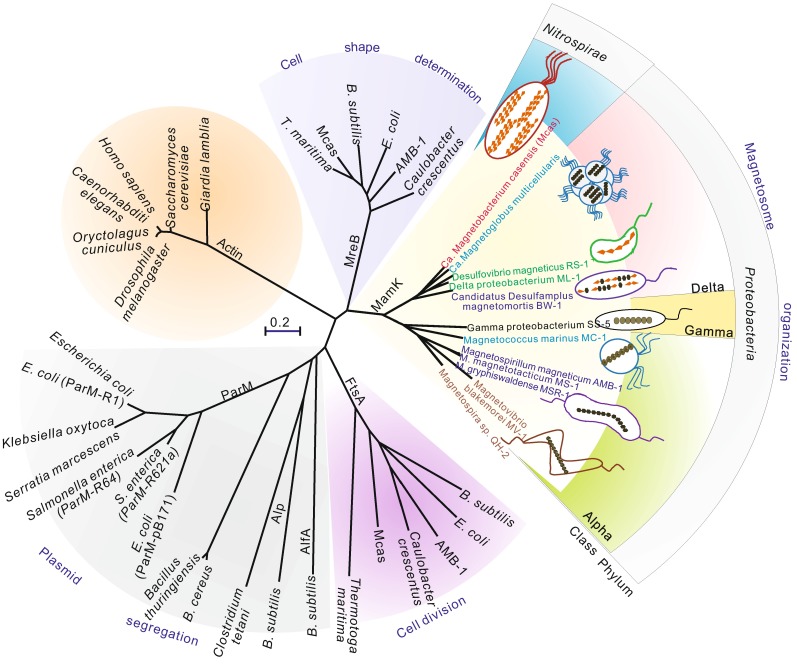
Phylogenetic tree based on amino acid sequences of actin-like and closely related proteins found in bacteria. The eukaryotic actin proteins from distinct sources including *Saccharomyces cerevisiae*, *Caenorhabditis elegans*, *Giardia lamblia*, *Drosophila melanogaster*, *Oryctolagus cuniculu* and *Homo sapiens* were used to root the tree. The MamK proteins from cultured and uncultured magnetotactic bacteria that have known magnetosome structures clustered into two groups. Accession numbers are given in Table S1. The tree is based on a maximum likelihood algorithm. The bar represents 20% sequence divergence

Cytoskeletal proteins have been identified in all organisms as a basic structural framework. They are critical for many important cellular processes, such as cell growth, shape maintenance, cell division, motility and internal transport pathways (Pollard and Cooper, 2009; Shih and Rothfield, 2006; Cabeen and Jacobs-Wagner, 2005, 2010). Among these proteins, actin is one of the most abundant and highly conserved proteins in most eukaryotic cells. Recent studies, however, have demonstrated that actins are also present in prokaryotic cells and have multiple functions ranging from the maintenance of cell shape to the organization of subcellular structures (Ozyamak et al., 2013a; Carballido, 2006). To date, approximately 40 different actin protein families sharing less than 30% identity have been discovered in prokaryotic organisms (Derman et al., 2009). Despite their low sequence identities, all bacterial actin proteins contain a similar tertiary structure and a common ATP-binding pocket (Carballido, 2006). However, unlike their eukaryotic counterparts bacterial actin proteins possess unique physical functions and biochemical and structural features (Ozyamak et al., 2013a). Moreover, bacterial actins are divided into distant families according to their physical functions, and the protein sequences of families from different sources are highly divergent. MreB, ParM, FtsA and MamK form four phylogenetically and functionally distinct groups of bacterial actin proteins and play vital roles in controlling cell shape (Reimold et al., 2013), segregating plasmid DNA (Garner et al., 2004), dividing cells (Szwedziak et al., 2012) and organizing the subcellular magnetosome chain structure (Komeili et al., 2006), respectively (Fig. 1).

Magnetosomes are intracellular nano-sized membrane-enveloped magnetite (Fe_3_O_4_) and/or greigite (Fe_3_S_4_) minerals that are normally organized into one or multiple chain structures in magnetotactic bacteria (MTB) (Bazylinski et al., 2013a). This chain arrangement confers the ability to sense the Earth’s magnetic field to MTB and facilitates the orientation of these bacteria to low-oxygen microhabitats (Faivre and Schüler, 2008). Magnetosome chains in MTB are attractive model systems for investigating the molecular mechanisms of organelle-like structure formation in prokaryotic cells (Schüler, 2008; Cornejo et al., 2014). The formation of magnetosomes is under strict genetic control through a group of magnetosome-associated proteins involved in magnetosome membrane biogenesis, magnetosome biomineralization and magnetosome membrane chain arrangement (Rahn and Komeili, 2013; Kolinko et al., 2014; Murat et al., 2010). Bacteria that form magnetosomes have been thus far detected in the *Alphaproteobacteria*, *Deltaproteobacteria* and *Gammaproteobacteria* classes of the *Proteobacteria* phylum, the phylum *Nitrospirae* and the candidate division OP3 (Lin et al., 2014a). It was recently reported that some members of the candidate phylum *Latescibacteria* have the genetic potential for greigite magnetosome formation (Lin and Pan, 2015). Magnetosomes in *Magnetospirillum* from the *Alphaproteobacteria* are flanked by a series of cytoskeletal filaments consisting of MamK proteins, which are assembled into a well-organized chain structure (Komeili et al., 2006; Katzmann et al., 2010; Draper et al., 2011). In the absence of MamK, the cytoskeletal filaments in *Magnetospirillum* disappear and the chain formation/structure changes. In *M. magneticum* AMB-1 (AMB-1), the *mamK* deletion mutant lacks the long and highly organized magnetosome chains observed in the wild-type strain (Komeili et al., 2006). The loss of *mamK* in *M*. *gryphiswaldense* MSR-1 (MSR-1) results in shorter, fragmented and ectopic chains (Katzmann et al., 2010). Moreover, MamK in MSR-1 plays a role in the magnetosome chain recruitment to the cell division site (Lin and Pan, 2011; Katzmann et al., 2011). Several studies have shown that MamK from *Magnetospirillum* forms filaments *in vitro* as well (Sonkaria et al., 2012; Taoka et al., 2007; Ozyamak et al., 2013b). These results suggest that MamK plays a crucial role in the formation and genetic stability of magnetosome chains.

To date, all known MamK proteins form two distinct groups (Fig. 1). One group includes MamK from the *Alphaproteobacteria* and *Gammaproteobacteria*, in which cuboidal/elongated magnetosomes are arranged into single chains in each cell (Lefèvre et al., 2012; Bazylinski et al., 2013b; Zhu et al., 2010; Bazylinski et al., 2013c; Lefèvre and Bazylinski, 2013). Among this group, the *in vitro* biochemical properties and assembly behaviours of MamK proteins from three closely related *Magnetospirillum* species have been extensively studied (Sonkaria et al., 2012; Taoka et al., 2007; Ozyamak et al., 2013b). They have nucleotide hydrolysis activities and assemble into filaments. However, MamK proteins from MSR-1 (Taoka et al., 2007), AMB-1 (Ozyamak et al., 2013b) and *M. magnetotacticum* MS-1 (MS-1) (Ozyamak et al., 2013b) have displayed diverse behaviours, including the role in nucleotides and filament assembly, which suggests a biochemical and functional variation in MamK proteins from different species.

MamK proteins from the *Deltaproteobacteria* and *Nitrospirae* phylum that arrange bullet-shaped or irregular magnetosomes into single or multiple chain(s) (Lefèvre and Wu, 2013; Jogler et al., 2011; Lin et al., 2014b; Lefèvre et al., 2011) clustered into another group (Fig. 1). Because more complex and intricate three-dimensional organizations of the multiple magnetosome strands are present in ‘*Candidatus* Magnetobacterium bavaricum’ (Mbav) (Jogler et al., 2011) and ‘*Ca.* Magnetobacterium casensis’ (Mcas) (Lin et al., 2014b) in the *Nitrospirae* phylum, the structure and function of the magnetosome cytoskeleton in these bacteria are speculated to be distinct from those of the MamK cytoskeleton in *Magnetospirillum* species. However, due to a lack of pure cultures, the current knowledge of biochemical properties of MamK proteins in the latter group remains very limited.

Recently, the draft genome of an uncultivated *Nitrospirae* MTB strain Mcas was recovered (Lin et al., 2014b). Mcas forms hundreds of bullet-shaped magnetite magnetosomes arranged into multiple bundles of chains within a single cell. The genome contains a magnetosome gene cluster with a complete *mamK* gene and other magnetosome-associated genes responsible for the formation of bullet-shaped multiple-chain magnetosomes. The MamK protein (GenBank accession AIM41328) in Mcas is ~40% identical to that of the *Magnetospirillum* species and contains the similar ATPase binding motifs conserved in all bacterial actin proteins (Lin et al., 2014b). In the present study, we characterized the *in vitro* biochemical properties and assembly behaviour of Mcas MamK.

## **RESULTS**

### **Nucleotide substrate preference and hydrolysis reaction processes of MamK**

**Figure 2:**
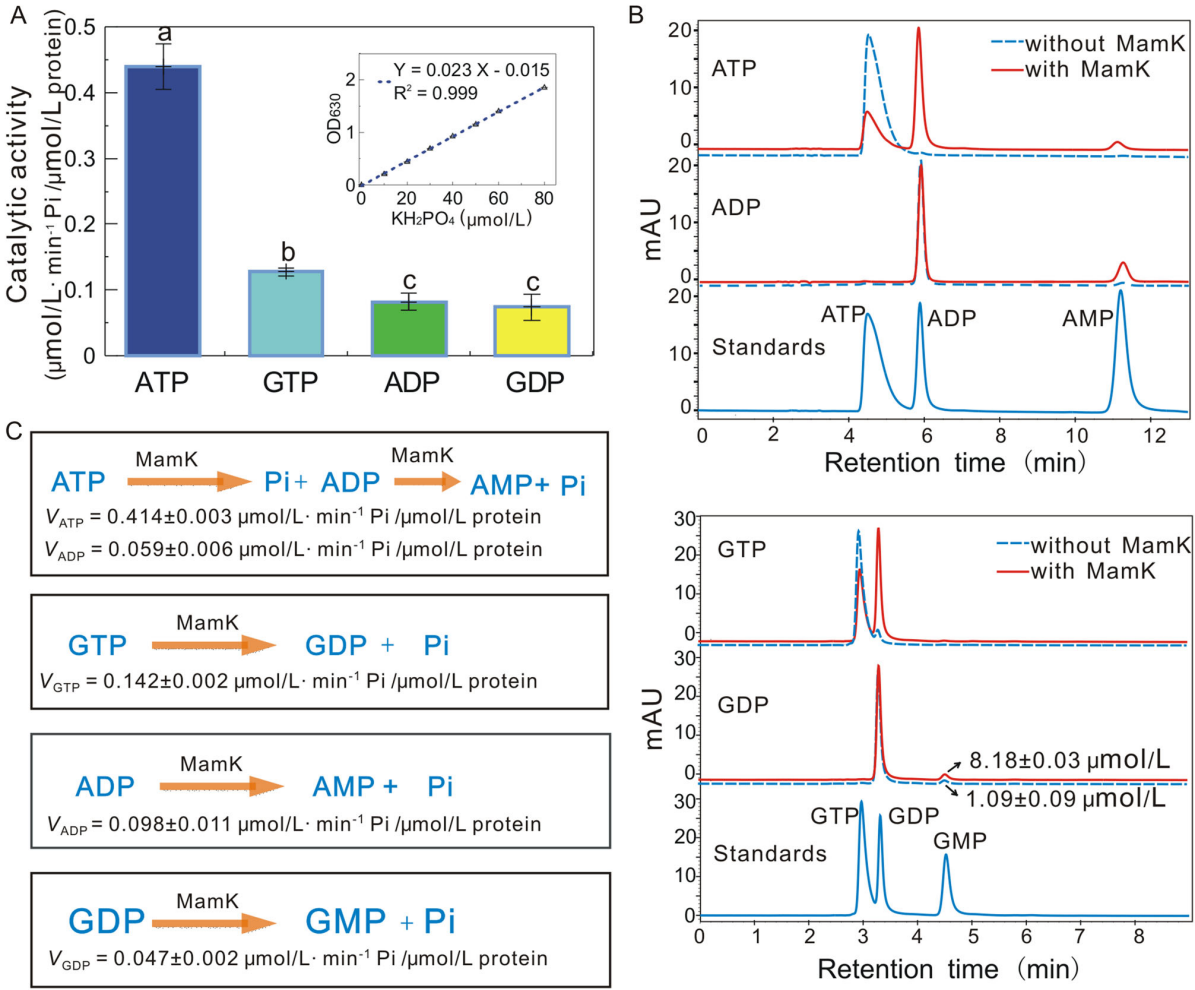
Catalytic efficiencies and reaction processes of MamK towards various nucleotide states. (A) Various NTPase and NDPase activities were assayed based on phosphate release. The inset shows the KH_2_PO_4_ standard curve from 0 to 80 μmol/L. MamK hydrolyzed ATP, GTP, ADP and GDP and showed the highest catalytic activity towards ATP. Results are given as the mean ± SD (*n* = 3). Different letters indicate significant differences among various nucleotide substrates (Duncan’s multiple range test, *P* < 0.05). (B) HPLC detection of the products formed from the hydrolysis of different nucleotide substrates by MamK. As a negative control, the reaction mixture was incubated under identical conditions, but in the absence of the MamK protein. ATP, ADP AMP, GTP, GDP and GMP were used as standards. (C) Catalytic reaction of MamK NTPases and NDPases. MamK catalyzed the formation of NDP and NMP from NTP and NDP, respectively

MamK from Mcas exhibited a broad substrate spectrum and could hydrolyze various nucleotides (ATP, GTP, ADP and GDP) and release Pi (Fig. 2A). It showed a high preference for ATP with a hydrolysis activity of 0.44 ± 0.04 μmol/L·min^−1^ Pi/μmol/L protein, which was 3-fold higher than that obtained with GTP. In the presence of ADP and GDP, however, low hydrolysis activities have been detected. The preferred order of MamK nucleotide hydrolysis activity was ATP > GTP > ADP (or GDP), suggesting that MamK preferentially hydrolyzes NTP over NDP.

The substrates and products of nucleotide hydrolysis were separated and quantified through high-performance liquid chromatography (HPLC) (Fig. 2B). In the presence of ATP or GTP, MamK could break phosphate bonds and generate ADP or GDP, respectively. These results demonstrate that the MamK protein converts ATP to ADP and GTP to GDP at rates of 0.414 ± 0.003 and 0.142 ± 0.002 μmol/L·min^−1^ Pi/μmol/L protein, respectively, consistent with the results of phosphate release assay shown in Fig. 2A. Trace AMP was produced through the additional hydrolysis of ADP (0.059 ± 0.006 μmol/L·min^−1^ Pi/μmol/L protein) by MamK protein (Fig. 2C). Similar to NTP hydrolysis, ADP and GDP were hydrolyzed by MamK and converted to AMP and GMP, respectively (Fig. 2B). The hydrolysis rates of ADP and GDP were calculated as 0.098 ± 0.011 and 0.047 ± 0.002 μmol/L·min^−1^ Pi/μmol/L protein, respectively (Fig. 2A and 2C). As observed by HPLC, GMP was produced by GDP hydrolysis in the absence of MamK. However, the hydrolysis velocity of GDP in the absence of MamK was approximately 0.04 μmol/L·min^−1^ of Pi, significantly lower than 0.27 μmol/L·min^−1^ of Pi in the presence of MamK.

### **Biochemical properties of MamK ATPase activity**

**Figure 3:**
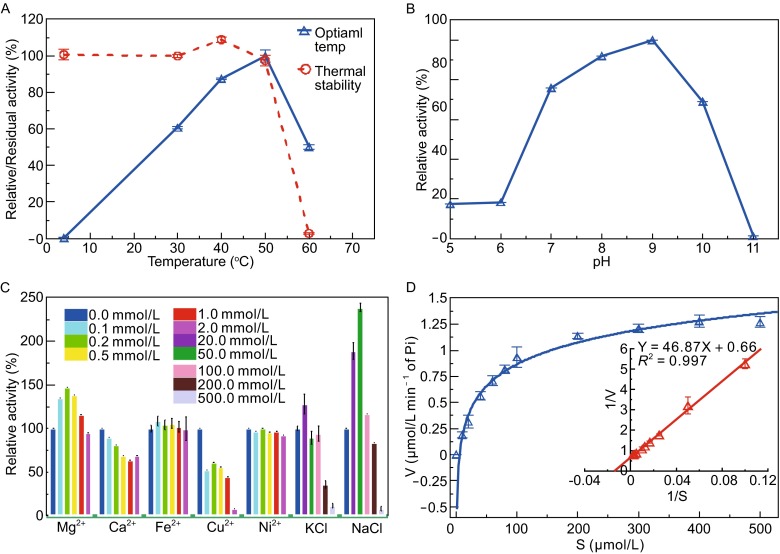
The biochemical properties of MamK ATPase activity. (A) The effect of temperature. The maximum enzyme activity and thermal stability were observed at 50°C and 40°C, respectively. (B) The effect of pH. More than 80% of MamK enzyme activity was retained within a pH range of 7.0–9.5, with maximum activity observed at pH 9.0. (C) The effects of various divalent cations and salts. Concentrations ranging from 0.1 to 1.0 mmol/L Mg^2+^ and from 20 to 100 mmol/L NaCl significantly affected MamK ATPase activity. (D) The kinetic parameters assayed under different concentrations of ATP. The Michaelis constants *Vmax* and *Km* for the enzyme reaction were determined as the reciprocal of the absolute x-intercept and y-intercept, respectively, after plotting the reciprocal of the catalytic efficiency (1/V) versus the reciprocal of the substrate concentration (1/S)

The effects of temperature, pH, divalent metal ions and salts on the ATPase activity of MamK have been investigated (Fig. 3). MamK could retain more than 80% activity at temperatures ranging from 35 to 55°C and a pH ranging from 7.0 to 9.5, with maximum enzyme activity observed at 50°C and pH 9.0, (Fig. 3A and 3B). To determine the tolerant ability of MamK protein towards high temperatures, the thermal stability was evaluated by its residual ATPase activity after incubation of MamK at temperatures ranging from 30°C to 70°C for 30 min. As shown in Fig. 3A, MamK retained appropriately 100% activities at the temperatures of 30°C–40°C, but the residual activity started to decrease at 50°C and could not be detected at 60°C, indicating that MamK protein was stable up to 40°C and started to denature around 50°C.

Among the various divalent metal ions and salt concentrations ranging from 0 to 500 mmol/L, 0.1–1.0 mmol/L of Mg^2+^ could activate MamK with maximum activity at 0.2 mmol/L, whereas inhibitory effects were observed at concentrations above 2.0 mmol/L (Fig. 3C). Nearly no effects on the enzyme’s activity were observed for Fe^2+^ or Ni^2+^ at concentrations of 0–2.0 mmol/L; however, inhibitory effects were observed for Ca^2+^ and Cu^2+^ at each concentration tested. The enzyme activity was slightly activated by 20 mmol/L KCl, but inhibition of enzyme activity increased with increasing salt concentrations. NaCl concentrations ranging from 20–100 mmol/L had a significant stimulatory effect on ATPase activity with a maximum relative activity of approximately 238.9% in the presence of 50 mmol/L NaCl (Fig. 3C). Therefore, Mg^2+^ and NaCl played roles in the activation of MamK ATPase activity. The optimal conditions for the ATPase activity of MamK were 0.2–1.0 mmol/L Mg^2+^, 20–100 mmol/L NaCl/20 mmol/L KCl, pH 7.0–9.0 and 35°C–50°C. The biochemical properties of MamK ATPase activity could provide a reference for investigating the physicochemical conditions of its polymerization.

The enzyme kinetic parameters were determined to further define the unique characteristics of the ATPase activity of MamK. Based on the plot shown in Fig. 3D, we calculated the *Vmax* and *Km* of the ATPase activity as 1.52 μmol/L·min^−1^ Pi/μmol/L protein and 71.02 μmol/L, respectively. The catalytic constant (*Kcat*) and efficiency (*Kcat*/*Km*) were calculated as 3.8 min^−1^ and 0.08 min^−1^·μmol/L^−1^, respectively.

### **MamK assembles into well-ordered filaments in an ATP-dependent manner**

**Figure 4:**
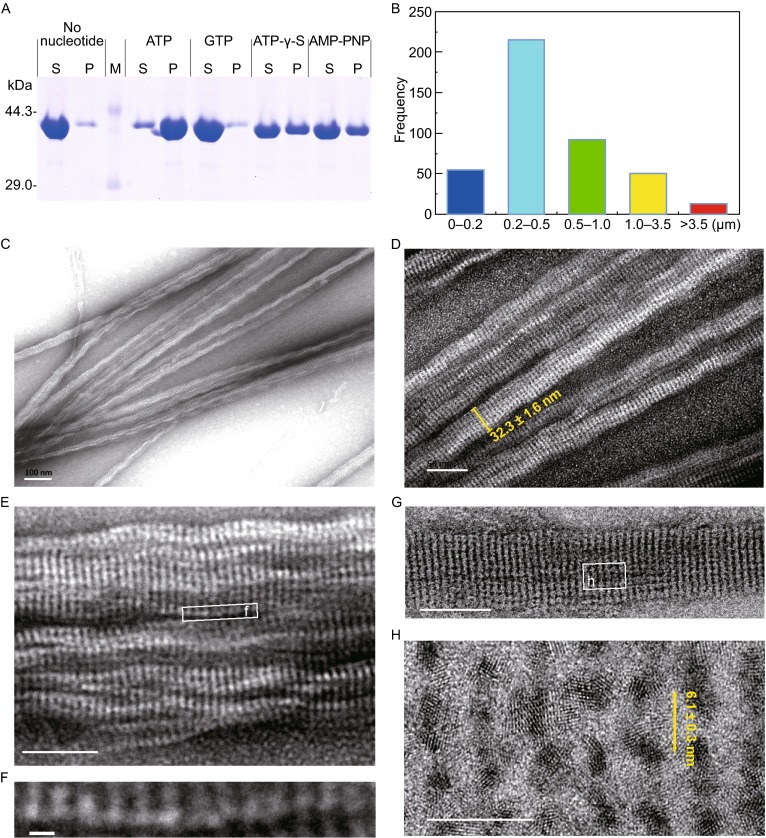
MamK filaments formed in the presence of ATP. (A) MamK polymerization in the presence of various nucleotide states was assessed by a pelleting assay. The supernatant (S) and pellet (P) fractions were analyzed by SDS-PAGE. In the presence of ATP, ATP-γ-S or AMP-PNP, the proportion of the pellet in the total protein was clearly increased compared to the negative control without any nucleotide. (B) The distribution of the filamentous lengths formed in the presence of ATP, Mg^2+^ and NaCl. The majority of MamK filaments ranged from 0.2 to 0.5 μm in length, and the well-developed filaments were more than 3.5 μm in length. (C–H) Negatively stained MamK polymers visualized by TEM. *In vitro*, MamK polymerized into well-ordered filaments comprised of multiple filaments in an ATP-dependent manner. Panel C scale bars, 100 nm; panel D scale bar, 50 nm; panel E scale bar, 50 nm; panel F scale bar, 5 nm; panel G scale bar, 50 nm; and panel H scale bar, 10 nm

Among the various parameters affecting bacterial actin polymerization, nucleotide substrates were crucial for the assembly of MamK into filaments. A pelleting assay was performed to determine the distribution of MamK between the monomeric and polymeric states after the MamK was purified (Fig. S1). The formation and structure of filaments *in vitro* were confirmed through transmission electron microscopy (TEM). In the absence of nucleotides, MamK proteins primarily remained in the soluble fraction and no filament formation was observed (Figs. 4A and S2A). Similar results were observed after the addition of GTP (Fig. 4A). In contrast, most of proteins were detected in pellet fraction in the presence of ATP. Moreover, the addition of nonhydrolyzable ATP analogues ATP-γ-S and AMP-PNP resulted in a smaller proportion of protein in the pellet fraction relative to the supernatant (Fig. 4A). However, filaments assembled with ATP-γ-S were loose and irregularly shaped, whereas filaments formed with AMP-PNP were shorter than those with ATP (Fig. S2C and S2D). When MamK assembled with ATP, well-ordered filaments with long and straight bundles comprising multiple filaments were observed. These well-developed filamentous bundles were greater than 3.5 μm in length and 30 nm in width (Fig. 4B and 4C). These bundles with average diameters of 32.3 ± 1.6 nm were highly ordered through multiple filaments with widths of approximately 6.1 ± 0.3 nm (Fig. 4D–H), which is different from the twisted filaments observed in the *Magnetospirillum* MamK bundles (Sonkaria et al., 2012; Taoka et al., 2007). These results indicate that Mcas MamK could polymerize into long, straight, well-ordered filaments *in vitro* in an ATP-dependent manner.

His-tags could affect the assembly behaviours of MamK and MreB homologs (Ozyamak et al., 2013b; Esue et al., 2005). In the present study, MamK with a poly-histidine tag at the C- or N-terminus was examined (Fig. S3). Compared to the well-ordered filamentous bundles polymerized by the MamK with three residues at the N-terminus after the poly-histidine tag being removed, his-tagged MamK formed shorter and irregular filaments under the same polymerization conditions (Fig. S3B and S3C). Therefore, his tags could influence the formation of Mcas MamK filamentous structures, likely through alterations in MamK polymerization.

### **MamK polymer formation under unique physicochemical conditions**

To further characterize the parameters affecting the ATPase activity of MamK, we have analyzed the effects of different physicochemical conditions (temperature, divalent cations and salts) on MamK polymerization. At temperatures ranging from 20°C–50°C, MamK assembled into regular filaments with the highest efficiency and the best-ordered structures observed at 37°C. MamK proteins denatured at temperatures above 50°C (Fig. S4). In the absence of metal ions, MamK proteins could assemble into well-ordered filaments with ATP, similar to the products obtained in the presence of both ATP and Mg^2+^. Mg^2+^ slightly increased the amount of filaments, suggesting that the polymerization rate of MamK could be improved by Mg^2+^ (Fig. S5A). Furthermore, the pelleting assay revealed that more filaments were formed in the presence of Ca^2+^ compared to Mg^2+^. However, in the presence of Ca^2+^ single disordered and irregularly shaped filaments were formed that lacked obvious bundles (Fig. S5E). These results suggest that the polymerization of MamK is independent of metal ions, but could be stimulated by Mg^2+^ and Ca^2+^. The filaments formed in the absence of metal ions or in the presence of Mg^2+^ were well ordered, whereas in the presence of Ca^2+^, the polymers were irregular.

**Figure 5:**
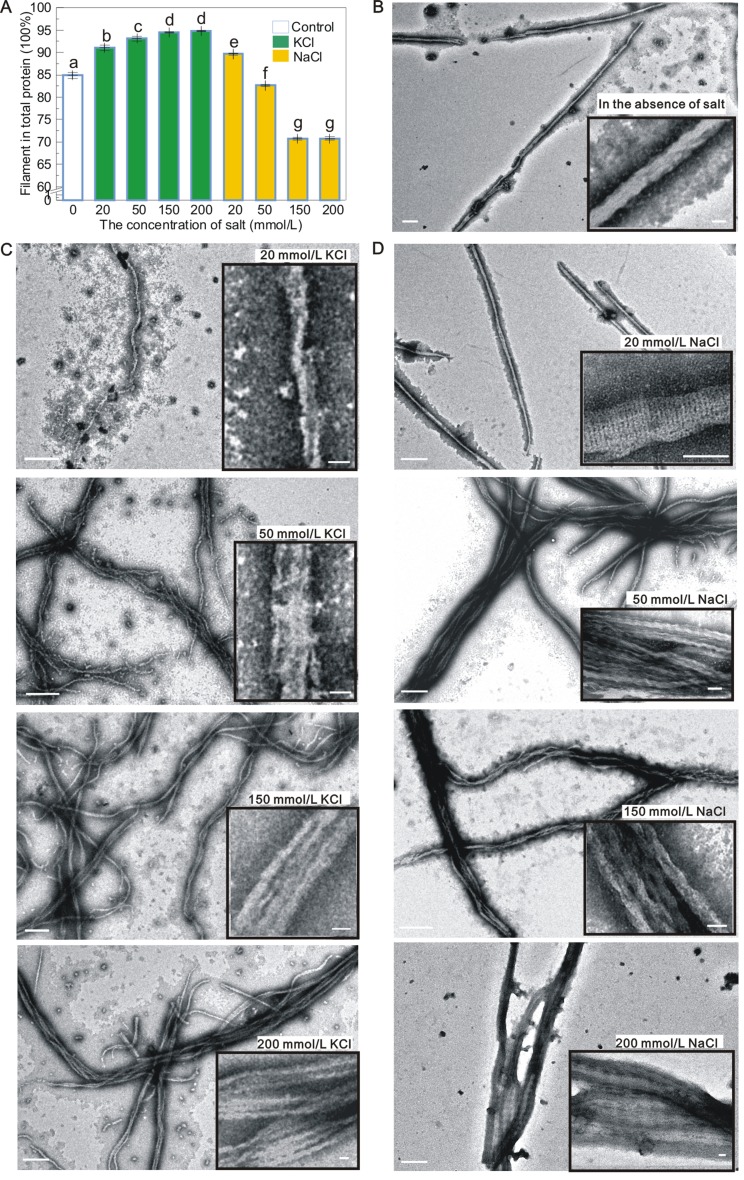
MamK polymers assembled into bundles in a salt-dependent manner. (A) The efficiency of MamK filament polymerization in different salt concentrations. The percent ratio of MamK filaments in the total protein represents the polymerization efficiency. Results are given as the mean ± SD (*n* = 4). Different letters indicate significant differences among the various concentrations of salt (Duncan’s multiple range test, *P* < 0.05). (B) Single MamK filaments were observed in the absence of salt. (C and D) MamK filaments formed in KCl and NaCl at concentrations ranging from 20 to 200 mmol/L. The formation of single filaments was observed in the presence of 20 mmol/L KCl or NaCl, and filaments with a well-ordered structure were observed with NaCl. MamK filaments preferentially clustered to form bundles in the presence of increasing KCl or NaCl concentrations ranging from 50 to 200 mmol/L. The insets show high magnification images. The 500 nm and 50 nm scale bars are shown in the low and high magnification images, respectively

Because KCl and NaCl are important in bacterial actin assembly, the effects of these cations on MamK assembly were also investigated (Fig. 5). The polymerization efficiency was slightly improved by increasing concentrations of KCl from 20–200 mmol/L; however, the efficiency decreased with increasing NaCl concentrations (Fig. 5A). The effects of salts on MamK assembly were different from the effects of those on ATPase activity (Fig. 3C). The filaments formed in the presence of different salt concentrations were further investigated through TEM. In the presence of 0–20 mmol/L KCl or NaCl, only single filaments were observed (Fig. 5B–D). The structures of filaments formed with NaCl were better organized than those formed with KCl. When the salt concentration was increased to 50 mmol/L, the filaments preferentially clustered to form multiple bundles. An increase in KCl or NaCl from 50–200 mmol/L induced the formation of multiple filament bundles, suggesting that the bundling of MamK filaments was dependent on the ionic strength of buffer.

### **MamK polymerization kinetics**

**Figure 6:**
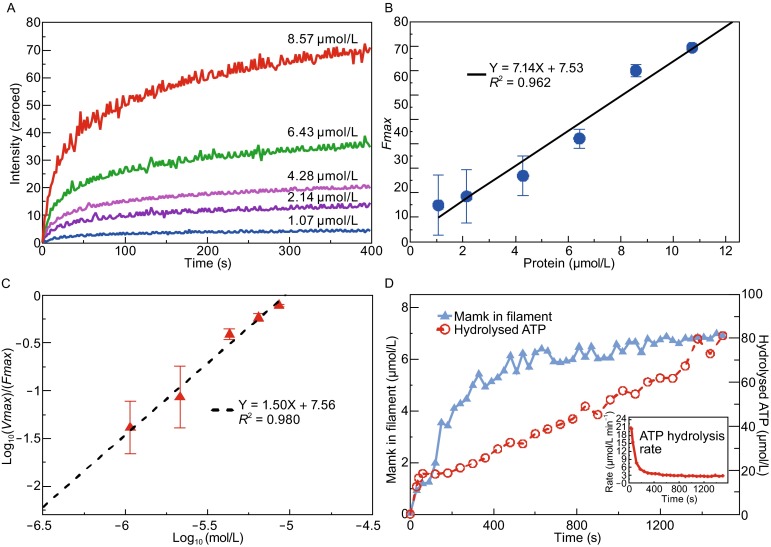
Polymerization kinetics of MamK. (A) Assembly kinetics of MamK at varying concentrations under standard conditions. (B) Determination of the critical concentration, which was determined as the x-intercept after plotting the maximum intensity values versus the protein concentration in (A). (C) Determination of the nucleus size and relative nucleation rates. The logarithm of the maximum polymerization rate is plotted versus the logarithm of the protein concentration in (A). The nucleus size (*n*) was estimated as two times the slope of the linear fit. The relative nucleation rate (*Kn*) was determined as the x-intercept in the plot. (D) Steady ATP hydrolysis during MamK polymerization. MamK in the filaments was determined as the total protein concentration minus the monomer concentration by the Bradford method after the monomers and filaments were separated. ATP hydrolysis was measured as the amount of phosphate released at various time points, and the inset shows the ATP hydrolysis rate (µmol/L·min^−1^ of Pi) defined as the amount of released phosphate per minute

The individual filaments of Alexa 488-labelled MamK polymerized in ATP were traced and imaged using time-lapse microscopy. We could not detect any growing filaments in any of the nucleotide substrates other than ATP. The filament structures formed by 0–8.57 μmol/L of labelled MamK were confirmed by TEM (Fig. S6). The detailed processes of MamK filament assembly in the presence of ATP were shown in Fig. S7. The polymerization kinetics of MamK filaments labelled with Alexa 488 was examined using increasing protein concentrations in saturating amounts of ATP. The polymerization rate initially increased in a protein concentration-dependent manner (Fig. 6A). The assembly kinetics was consistent with a cooperative polymerization mechanism comprised of two apparent phases: an initial increase in polymer formation followed by a slow approach to steady state. A long lag phase was reached during protein polymerization at 1.07 μmol/L. Polymer formation below 1.0 μmol/L of MamK protein could not be detected by fluorescence microscopy. The critical concentration of filament assembly in the presence of Mg^2+^-ATP and NaCl was calculated as 1.0 μmol/L (Fig. 6B), a value greater than that observed for MamK from AMB-1 (~0.7 µmol/L) (Ozyamak et al., 2013b) but less than that for MamK from MSR-1 (1.4 µmol/L) (Sonkaria et al., 2012). A plot of the logarithm of maximum polymerization rate versus the logarithm of protein concentration revealed that the nucleus size of MamK filament was trimeric in Mg^2+^-ATP and NaCl (Fig. 6C). The rate constant of nucleation reaction was 5.04, reflecting a rapid nucleation rate similar to ParM (~4.82–4.84) (Garner et al., 2004; Rivera et al., 2011).

As shown in Fig. 6D, ATP hydrolysis through MamK lagged behind ATP polymerization, except during the initial reaction period (0–60 s), in which they appeared to be concomitant. In the initial nucleation phase, there was rapid formation of nucleotide triphosphate-bound MamK monomers, which had undergone rapid hydrolysis, for the nucleus establishment. Subsequently, the ATP hydrolysis rate decreased sharply and a constant relative rate of phosphate production (3.2 ± 0.2 μmol/L·min^−1^ of Pi) was observed at both increasing and steady states of polymerization. Therefore, it is reasonable to speculate that ATP hydrolysis is triggered through MamK polymerization and might play an essential role in filament turnover. According to the results of the present study, a model for the filament assembly of MamK in genus ‘*Ca.* magnetobacterium’ could be proposed (Fig. S8).

### **DISCUSSION**

Bacterial actin protein MamK plays an essential role in the linear chain arrangement of intracellular magnetite or greigite magnetosomes in MTB, which are a model system for investigating organelle biogenesis in prokaryotes (Schüler, 2008; Cornejo et al., 2014). In the present study, we have characterized the *in vitro* biochemical functions, structural features and assembly behaviours of MamK from Mcas, an uncultivated *Nitrospirae* MTB that forms multiple bundles of bullet-shaped magnetite magnetosome chains.

The hydrolysis of nucleotide phosphate is a common property of functionally diverse bacterial actin proteins. Although some bacterial actin proteins produce inorganic phosphates only in the presence of tri-phosphate nucleotides (Sonkaria et al., 2012; Mayer and Amann, 2009), MamK protein from Mcas produces phosphates not only in the presence of ATP and GTP but also with ADP and GDP (Fig. 2). In MSR-1, the levels of ATPase and GTPase activities for MamK were similar (Sonkaria et al., 2012); however, MamK from Mcas displayed a high ATPase activity (0.44 ± 0.04 μmol/L·min^−1^ Pi/μmol/L protein) that was three-fold higher than the GTPase activity. The ATPase activity of MamK from Mcas was higher than that from AMB-1 (Ozyamak et al., 2013b) but lower than that of ParM (approximately 3–6 μmol/L·min^−1^ Pi/μmol/L protein) (Garner et al., 2004; Rivera et al., 2011). Although Mcas MamK could bind and hydrolyze ADP and GDP, the catalytic activities towards both substrates were significantly lower than that towards ATP or GTP, with ATP as the most preferred substrate. We have developed an HPLC assay to further determine the hydrolysis of various nucleotides. The transitions of various NTPase and NDPase catalytic reactions between substrates and products were precisely determined, from which the MamK protein could convert NTP to NDP and NDP to NMP, respectively.

Previous studies have demonstrated the transitions of bacterial actins between monomeric and filamentous states (Ozyamak et al., 2013a). For instance, MamK proteins from MS-1 and MSR-1 polymerize into filaments *in vitro* with a nonhydrolyzable ATP analogue (ATP-γ-S) as the optimal substrate (Sonkaria et al., 2012; Taoka et al., 2007). In contrast, AMB-1 MamK with a His tag polymerized in the absence of nucleotides (Rioux et al., 2010), but untagged proteins efficiently assembled into filaments in the presence of ATP (Ozyamak et al., 2013b). Because the MamK proteins investigated in previous studies were more than 94% identical, the his tags used in different studies could alter the assembly behaviours of MamK proteins (Ozyamak et al., 2013b). Although Mcas MamK has a relatively low identity (~40%) to *Magnetospirillum* MamK, TEM observations showed that tags could significantly affect Mcas MamK filamentous structures (Fig. S3). Different assembly properties have also been observed for untagged and his-tagged MreB from *T. maritima* (Bean and Amann, 2008). Additionally, despite the broad hydrolysis of various nucleotides, Mcas MamK only assembled well in the presence of ATP. No filament was detected with GTP, and only irregularly shaped filaments were formed with nonhydrolyzable ATP analogues (ATP-γ-S or AMP-PNP) (Fig. S2). However, many bacterial actins, including ParM (Rivera et al., 2011), MreB (Mayer and Amann, 2009; Ent et al., 2001) and AlfA (Popp et al., 2010), could polymerize with ATP, GTP or analogues. Indeed, recent studies have revealed that AMB-1 MamK polymerizes in the presence of ATP and GTP at different critical concentrations (Ozyamak et al., 2013b). However, the efficient polymerization of regular Mcas MamK filaments was only triggered through ATP *in vitro*.

Certain physicochemical parameters including divalent cations and salts are important factors for bacterial actin protein assembly. Although the polymerization of AMB-1 (Ozyamak et al., 2013b) and MSR-1 (Sonkaria et al., 2012) MamK proteins requires Mg^2+^, this divalent cation was not strictly required for the assembly of Mcas MamK, though its polymerization rate was slightly enhanced by Mg^2+^. Previous studies have also suggested that Mg^2+^ was not necessary for the assembly of *Tm*MreB, and the inhibitory effect of this cation on *Tm*MreB was in contrast to that on MamK proteins (Esue et al., 2005; Ent et al., 2001). Sonkaria et al. monitored the light scattering intensity and revealed that 1 mmol/L of Ca^2+^ could maximize the polymerization efficiency of MSR-1 MamK (Sonkaria et al., 2012). A similar result was also observed in the present study, but the filamentous structure of Mcas MamK was irregular (Fig. S5). These results suggest that using light scattering intensity and pelleting assays to score bacterial actin polymerization should be cautious and needs to be further confirmed by transmission electron microscope observations.

Notably, salt concentrations strongly influence MamK polymerization. Sonkaria et al. reported that MamK from MSR-1 effectively polymerized in the presence of KCl at concentrations from 0–20 mmol/L, whereas polymerization was inhibited by increasing salt concentrations and the polymerization rate was drastically reduced by 200 mmol/L KCl (Sonkaria et al., 2012). In contrast, MamK from AMB-1 polymerized at high concentrations of KCl, and efficient assembly into bundles was observed at 150 mmol/L KCl (Ozyamak et al., 2013b). This KCl-dependent bundling was observed for Mcas MamK in this study. However, the bundling was initiated at 50 mmol/L KCl for Mcas MamK filaments, which is higher than that for AMB-1 MamK (Ozyamak et al., 2013b). Similar NaCl-dependent bundling was also observed for Mcas MamK, except that the efficiency of assembly decreased slightly with increasing salt concentrations from 20–200 mmol/L. Altogether, the polymerization conditions for different MamK proteins exhibited a surprising degree of variation for salts. The assembly of bundles might be an intrinsic property of bacterial actin because potassium and sodium are the most abundant cations in bacterial cells. Thus, this filament bundling at physiological K^+^/Na^+^ concentrations *in vitro* might be related to the static nature of MamK filaments in cells.

Considering the basic physicochemical parameters that define the polymerization of MamK, the assembly kinetics and nucleation mechanisms should be addressed. The molecular mechanism for MamK assembly remains largely unknown because it is difficult to detect assembly events like those of ParM (Garner et al., 2004; Rivera et al., 2011). We investigated the assembly behaviours of MamK filaments using fluorescence microscopy to examine Mcas MamK polymers *in vitro*. The bulk assembly kinetics of Mcas MamK displayed an initial peak in polymer concentration, followed by a slow approach to equilibrium (Fig. 6). In the initial assembly phase, a large number of dotted polymers formed with a high rate of ATPase activity, indicating that the early assembly of MamK proteins might create a burst of hydrolysis equal to the amount of MamK polymerized (Fig. S7). As the dotted polymers started to elongate and associate into bundles, the ATPase activity of MamK reached a plateau because of the limited subunit exchange for dissociation to exchange ADP for ATP and subsequent re-association for hydrolysis. Mcas MamK elongated from a nucleus comprised of three monomers, which is consistent with the nucleus size of AMB-1 MamK and ParM as previously suggested (Garner et al., 2004; Ozyamak et al., 2013b).

The divergent assembly of bacterial actin proteins might reflect the specific adaptation to distinct intracellular physiological functions. For example, the dynamic instability and bidirectional polymerization of ParM could provide the force needed to push plasmids to opposite poles of the cell (Garner et al., 2004; Fusinita et al., 2002). The polymerization of MamK should contribute to organize and maintain magnetosome chain structures. Previous studies have suggested that MamK might interact with methyl-accepting chemotaxis proteins (MCP) at cell poles (Philippe and Wu, 2010). According to our results, it is speculated that MamK monomers in Mcas might bind to ATP, form a three-monomer nucleus and further elongate to form filaments (Fig. S8). Further investigations are needed to fully understand the mechanisms of multiple magnetosome chain assembly in Mcas and their relatives belonging to the *Nitrospirae* phylum.

In summary, the present study provides evidence for the stable assembly of magnetosome-arranging protein MamK from Mcas that harbours multiple intracellular magnetosome chains. These results provide insights into the biochemical functions, assembly behaviour and filamentous structure of MamK in the poorly understood *Nitrospirae* phylum.

## **MATERIALS AND METHODS**

### **Cloning, expression and purification**

The *mamK* gene was amplified from Mcas genomic DNA, cloned into the pET28a and pET22b vectors and then expressed in *Escherichia coli* C43 (DE3) cells (Seignovert et al., 2004). The cells were grown in Luria-Bertani (LB) medium at 37°C and induced for 4 h with 0.5 mmol/L IPTG when the OD_600_ reached 0.8. Cells were harvested and disrupted through sonication in buffer A containing 20 mmol/L Tris-HCl (pH 8.0), 150 mmol/L NaCl and 10% glycerol. After centrifugation at 15,000 ×*g* for 30 min, MamK protein in the supernatant was purified through Ni^2+^ affinity chromatography and gel filtration. The detailed cloning, expression and purification procedures are described in the supplementary information. The purity of the sample was assessed through sodium dodecyl sulfate polyacrylamide gel electrophoresis (SDS-PAGE) (Fig. S1).

### **Fluorescent labelling of MamK**

For the labelling reactions, purified monomeric MamK was added to a 5-fold molar excess of Alexa-488 dye (Sigma, USA) and incubated at room temperature for 2 h. The reaction was quenched by the addition of 10 mmol/L DTT after the dye had reacted efficiently with the primary amines of proteins to form stable dye-protein conjugates. The labelled protein was purified through gel filtration on Bio-Rad BioGel P-30 fine size exclusion resin to remove the free dye. The degree of labelling is described in the supplementary information. The proteins labelled with approximately 1 mole of fluorophore per mole of MamK were used in the present study. The samples were divided into small aliquots, light protected and stored at −20°C. The ATPase activity and filamentous structure of the labelled proteins were determined to estimate their functions (Fig. S6).

### **Nucleotide hydrolysis activities assay**

A modified malachite-green method based on spectrophotometry was used to determine the NTPase and NDPase activities (Webb, 1992; Fisher and Higgins, 1994). The nucleotide hydrolysis activity was assayed in buffer B containing 20 mmol/L Tris-HCl (pH 8.0), 0.2 mmol/L MgCl_2_, 50 mmol/L NaCl and 0.2 mmol/L nucleotide substrate (ATP, GTP, ADP or GDP). The enzyme solution was added to a final concentration of 0.4 μmol/L in the total reaction mixture to determine the release of inorganic phosphate (Pi). The nucleotide hydrolysis reaction containing 2 μmol/L of MamK in buffer B was performed at 37°C for 30 min and then terminated after the addition of 4 volumes of chromogenic reagent, which was optimized as 0.67 mol/L H_2_SO_4_, 0.02 mol/L ammonium molybdate, 0.63% Tween 20 and 0.03% malachite green. The chromogenic reaction was performed at room temperature for 30 min, and the absorbance of the total reaction mixture was subsequently measured at OD_630_. The reaction mixture in the absence of enzyme treated under the same conditions was used as the control. The enzyme activity was defined as the amount of released phosphate (μmol/L) catalyzed by 1 μmol/L of enzyme per minute under the experimental conditions (Ozyamak et al., 2013b). The absorbance of the reaction mixture at 0 min and 30 min was assayed to calculate the released phosphate, which was quantified using a KH_2_PO_4_ standard curve. Based on the malachite-green assay, the MamK ATPase features were determined as described in the supplementary information. All measurements were performed in at least triplicate.

### **HPLC assay for nucleotide states**

The substrates and products of the NTPase and NDPase catalytic reactions were assayed by HPLC. After incubation at 37°C for 30 min, the assay was terminated upon the addition of 1 μL of 100% trichloroacetic acid (TCA). The samples were filtered through a 0.22-μm filter and ran on an Agilent 1200 HPLC equipped with a Zorbax SB-Aq column (4.6 × 250 mm; Agilent Technologies, USA). A 0.5% KH_2_PO_4_ solution (pH 4.5) and methanol were used as mobile phases A and C, respectively. HPLC was performed at a constant flow rate of 1.0 mL/min with 95% (*v*/*v*) phase A and 5% (*v*/*v*) phase C. The UV absorption was measured at 254 nm, and the column temperature was maintained at 25°C. Various nucleotide states, including ATP, ADP, AMP, GTP, GDP and GMP (Sigma, USA), were used as standards to determine the retention times and amounts of the various ingredients in the reaction mixture. The reaction mixtures assayed using the same procedure in the absence of MamK proteins served as negative controls. Compared with the negative controls, the hydrolysis rate was calculated by the change in the concentrations (μmol/L) of the substrates or products catalyzed by 1 μmol/L of enzyme per minute under the experimental conditions.

### **MamK polymerization and pelleting assays**

Purified MamK was added at a final concentration of 8.5 μmol/L in a total volume of 100 μL of polymerization buffer containing 20 mmol/L Tris-HCl (pH 8.0), 1 mmol/L MgCl_2_ and 50 mmol/L NaCl. A similar polymerization reaction without nucleotides was used as the negative control to exclude the aggregated protein. Various parameters affecting MamK assembly were determined as described in the supplementary information. The mixture was subsequently centrifuged at 500,000 ×*g* for 30 min to remove the aggregates. To polymerize the protein, ATP or other nucleotide states were added at a final concentration of 1 mmol/L, and the reaction mixture was immediately incubated at 37°C for 30 min. Steady state monomers and filaments were separated through centrifugation at 500,000 ×*g* for 30 min. The concentration of the protein monomers in the supernatant was rapidly determined using the Bradford method (Bio-Rad Protein Assay Dye Reagent, USA). To evaluate MamK polymerization, the pellets were suspended in 100 μL of polymerization buffer, and equal volumes of the supernatant and pellet samples were separated by SDS-PAGE on 5% stacking and 10% running gels. The protein bands were visualized by Coomassie Brilliant Blue G250 staining and quantitated using the NIH ImageJ program (http://rsb.info.nih.gov/ij/). The amount of pellets in the total protein was quantitated and verified as the amount of the monomer in the same reaction. MamK protein in the filament was calculated as the amount of pellets in the reaction mixture minus that of the negative control without nucleotide.

### **Transmission electron microscopy and image analysis**

MamK filaments were generated in the polymerization reaction mixture containing 20 mmol/L Tris-HCl (pH 8.0), 50 mmol/L NaCl, 1 mmol/L MgCl_2_, 8.5 μmol/L of protein and 1 mmol/L ATP. The mixtures were incubated at 37°C for 30 min and loaded onto carbon-coated grids for 5 min. The grids were washed twice with deionized water, followed by negative staining with 1% uranyl acetate for 1 min. The products on the grids were observed using a JEM-1400 transmission electron microscope (TEM) operating at 80 kV (JEOL Corporation, Japan). Images were captured by a Gatan 832 CCD camera (4,008 × 2,672 pixels). Protein samples incubated under the same polymerization conditions without substrate were used to confirm the absence of filamentous structures in the purified protein and the polymerization mixture without nucleotide substrate. Filament lengths of more than 400 polymers were analyzed using the NIH ImageJ program.

### **Polymerization kinetics, critical concentration and phosphate release**

Filament assembly was conducted using a fluorescence-based assay as described previously (Garner et al., 2004; Sahoo et al., 2006; Fujiwara et al., 2007). To prepare the polymerization samples, unlabelled protein was doped with 15% labelled MamK in polymerization buffer. The labelled proteins had absorption and fluorescence emission maxima of approximately 494 nm and 519 nm, respectively. The protein concentration and doping percentage were confirmed after measuring the absorbance at 280, 494 and 519 nm. Polymerization was indicated as the changes in the fluorescence intensity of the reaction mixture recording by a Zeiss Axioskop microscope using EMCCD camera (at an excitation wavelength of 493 nm and an emission wavelength of 517 nm) and ZEN image analysis system. The zero value of the fluorescence intensity was taken as the fluorescence of the reaction mixture in the absence of ATP. For the kinetic assays, MamK was added at the designed concentrations to the reaction buffer in a total volume of 10 μL. The protein concentration range was optimized at 1–10 μmol/L, at which the polymerization efficiencies clearly varied and the protein was stable and not prone to aggregation. As described previously (Nurse and Marians, 2013), the maximum intensity values were plotted versus the protein concentration to determine the critical concentration (the x-intercept). To calculate the nucleus size and nucleation rate, the logarithm of the maximum rate of polymerization was plotted versus the logarithm of the protein concentration. The nucleus size (*n*) was estimated as two times the slope of the linear fit, and the x-intercept was estimated as the relative nucleation rate (*Kn*) (Garner et al., 2004; Rivera et al., 2011). The error bars, which represent the average and standard deviations, were obtained by detecting the maximum polymerization rate in triplicate for each protein concentration. Because the ATPase activity of MamK was inhibited at 4°C (Fig. 3A), polymerization was assessed using a pelleting assay as described above. The amount of inorganic phosphate (Pi) released was quantified using a modified malachite-green method. All measurements were performed at least in triplicate, and the standard deviations (SD) were calculated from three independent experiments.

### **Phylogenetic analysis**

Amino acid sequences of various eukaryotic and prokaryotic actins were retrieved from the GenBank databases. Their sources and accession numbers are listed in Table S1. Sequence alignment was conducted using ClustalW, and a maximum-likelihood tree with 100 bootstrap replicates was constructed using MEGA version 4.0 (Tamura et al., 2007).

## Electronic supplementary material

Below is the link to the electronic supplementary material.

Supplementary material 1 (PDF 1518 kb)

Supplementary material 2 (PDF 378 kb)

Supplementary material 3 (PDF 128 kb)
