# Supplementary material for: In vitroassembly of the bacterial actin protein MamK from ‘CandidatusMagnetobacterium casensis’ in the phylumNitrospirae
Source: Protein Cell. 2016 Mar 9;7(4):267–80. doi: 10.1007/s13238-016-0253-x (PMC4818849; doi:10.1007/s13238-016-0253-x)
Supplement: Supplementary file 1 — Supplementary material 1 (PDF 1518 kb) [file 13238_2016_253_MOESM1_ESM.pdf]

Supplemental Figures

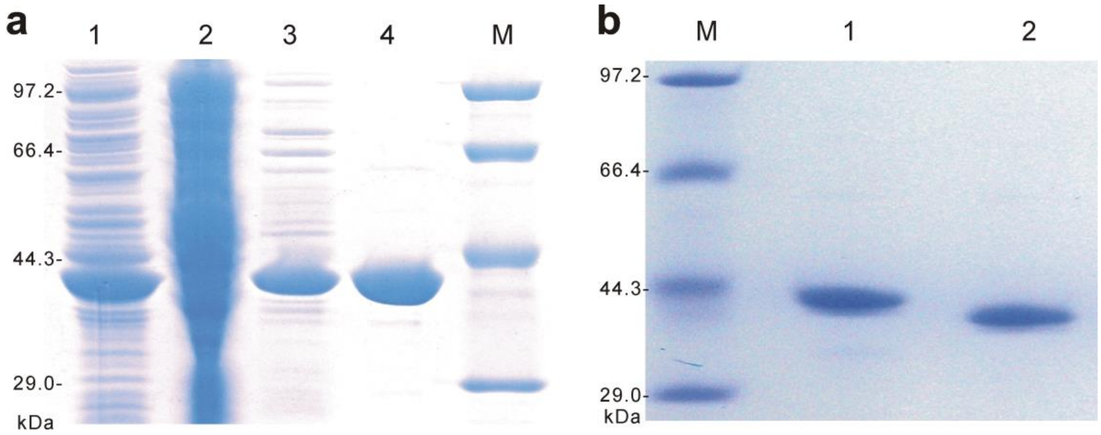

Supplementary Figure S1 | Purification of MamK. (a) Heterologous expression of the *mamK* gene and protein purification. Lane 1, *mamK* gene expressed in *E. coli* C43; Lane 2, unbound proteins in the flow-through fraction during Ni^{2+} affinity purification; Lane 3, his-tagged MamK protein purified by Ni^{2+} affinity chromatography; Lane 4, his-tagged MamK protein purified by gel filtration; Lane 5, low-molecular-weight markers. (b) Purification of MamK without his tags. Lane 1, low-molecular-weight markers; Lane 2, purified his-tagged proteins (41.5 kDa); and Lane 3, purified protein with three residues at the N-terminus (39.4 kDa).

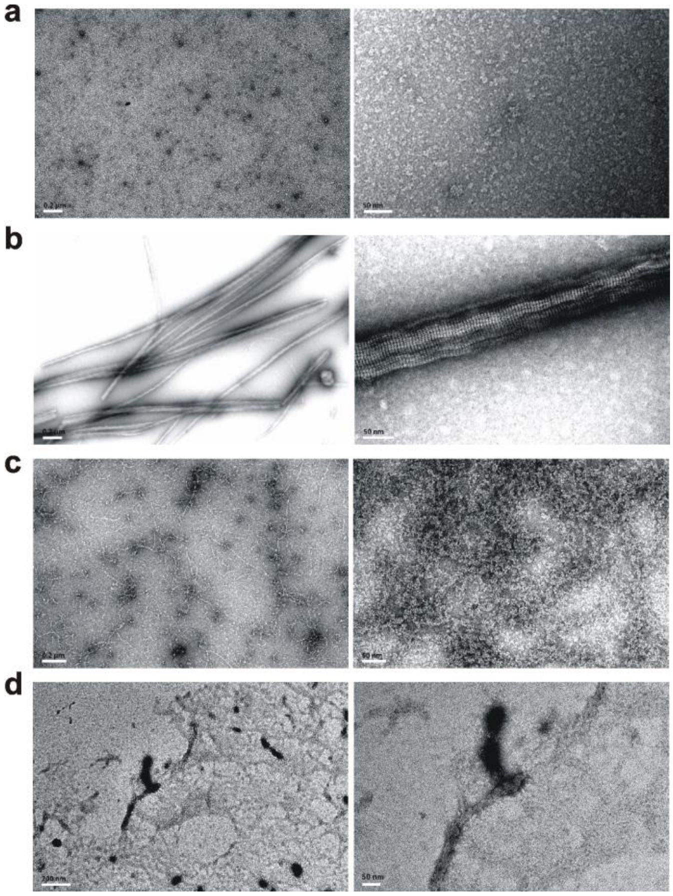

Supplementary Figure S2 | MamK filamentous structures formed in the presence of various nucleotide substrates. (a) No filaments were observed on the grids in the absence of nucleotides. (b) Well-ordered filaments formed in the presence of ATP. (c) Loose and irregularly shaped filaments assembled with ATP-γ-S. (d) Short and small filaments formed with AMP-PNP. The scale bars for the left and right images are 200 and 50 nm, respectively.

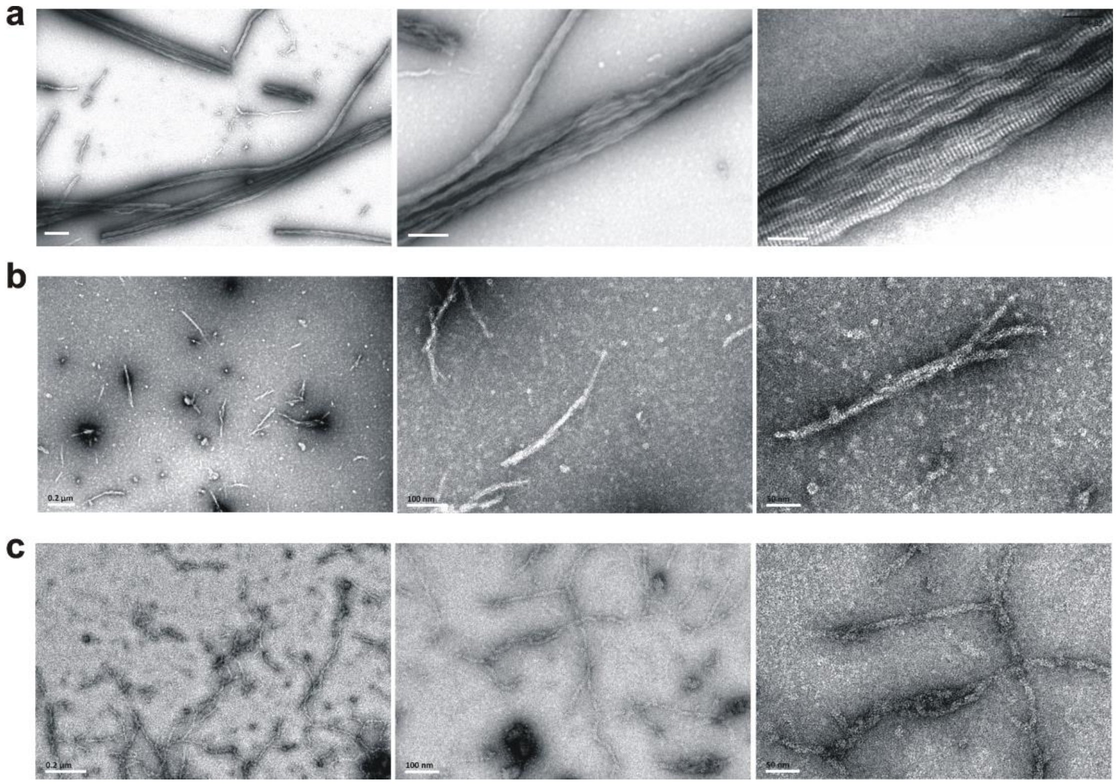

Supplementary Figure S3 | The effects of his tags on the formation of MamK filamentous structures in the presence of ATP. (a) Well-organized filamentous bundles formed with MamK proteins witout his tags. (b) Small and short filaments formed with his-tagged proteins at the N terminus. (c) Scattered and irregular filaments formed with his-tagged proteins at the C terminus. The scale bars for the left, middle and right images are 200, 100 and 50 nm, respectively.

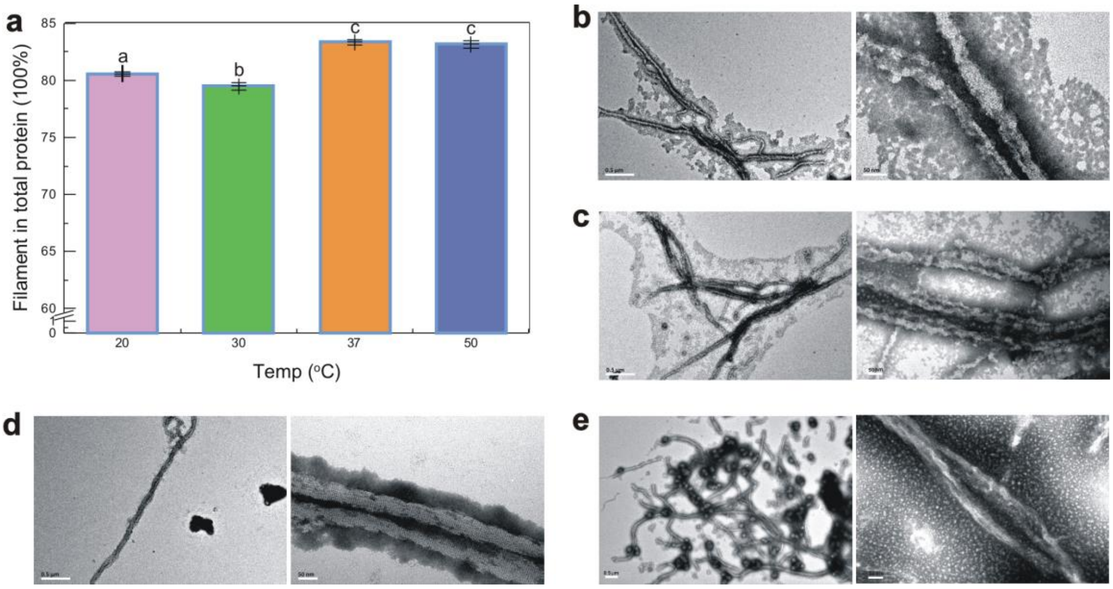

Supplementary Figure S4 | MamK polymerization at various temperatures. (a) The polymerization efficiency was assessed by a pelleting assay. MamK assembled into regular filaments with relatively high efficiency at temperatures of 37 ℃ and 50 ℃. Results are given as the means ± SDs (n=5). Different letters indicate significant differences among the various temperatures (Duncan's multiple range test, $p<0.05$). (b) Filamentous bundles formed at 20 ℃. (c) Filamentous bundles formed at 30 ℃. (d) Well-organized filamentous bundles formed at 37 ℃. (e) Filamentous bundles and many denatured proteins formed at 50 ℃. The standard polymerization conditions are described in the 'Methods' section. The scale bars for the left and right images are 500 and 50 nm, respectively.

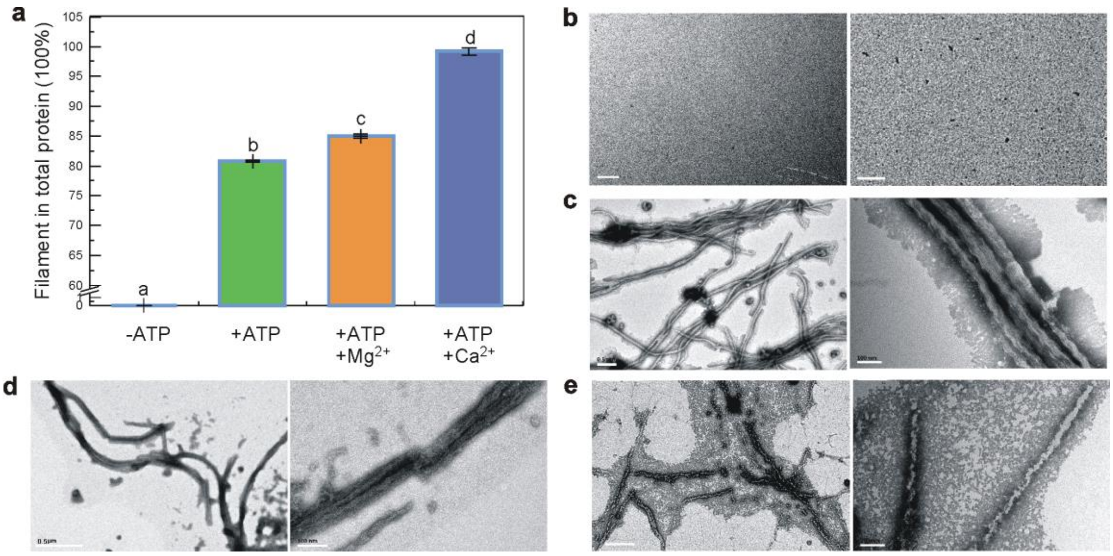

Supplementary Figure S5 | The effects of divalent cations on the formation of MamK filamentous structures. (a) The polymerization efficiency was assessed by the pelleting assay. The polymerization rate improved with Mg^{2+} or Ca^{2+} in the presence of ATP. Results are given as the means ± SDs (n=5). Different letters indicate significant differences among the various conditions (Duncan's multiple range test, $p<0.05$). (b) No filaments were observed on the grids in the absence of ATP. (c) Well-ordered filamentous bundles formed under standard conditions in the absence of divalent cations. (d) Well-ordered filamentous bundles formed under standard conditions in the presence of Mg^{2+}. (e) Single and irregularly shaped filaments formed under standard conditions in the presence of Ca^{2+}. The standard polymerization conditions are described in the 'Methods' section. The scale bars for the left and right images are 500 and 100 nm, respectively.

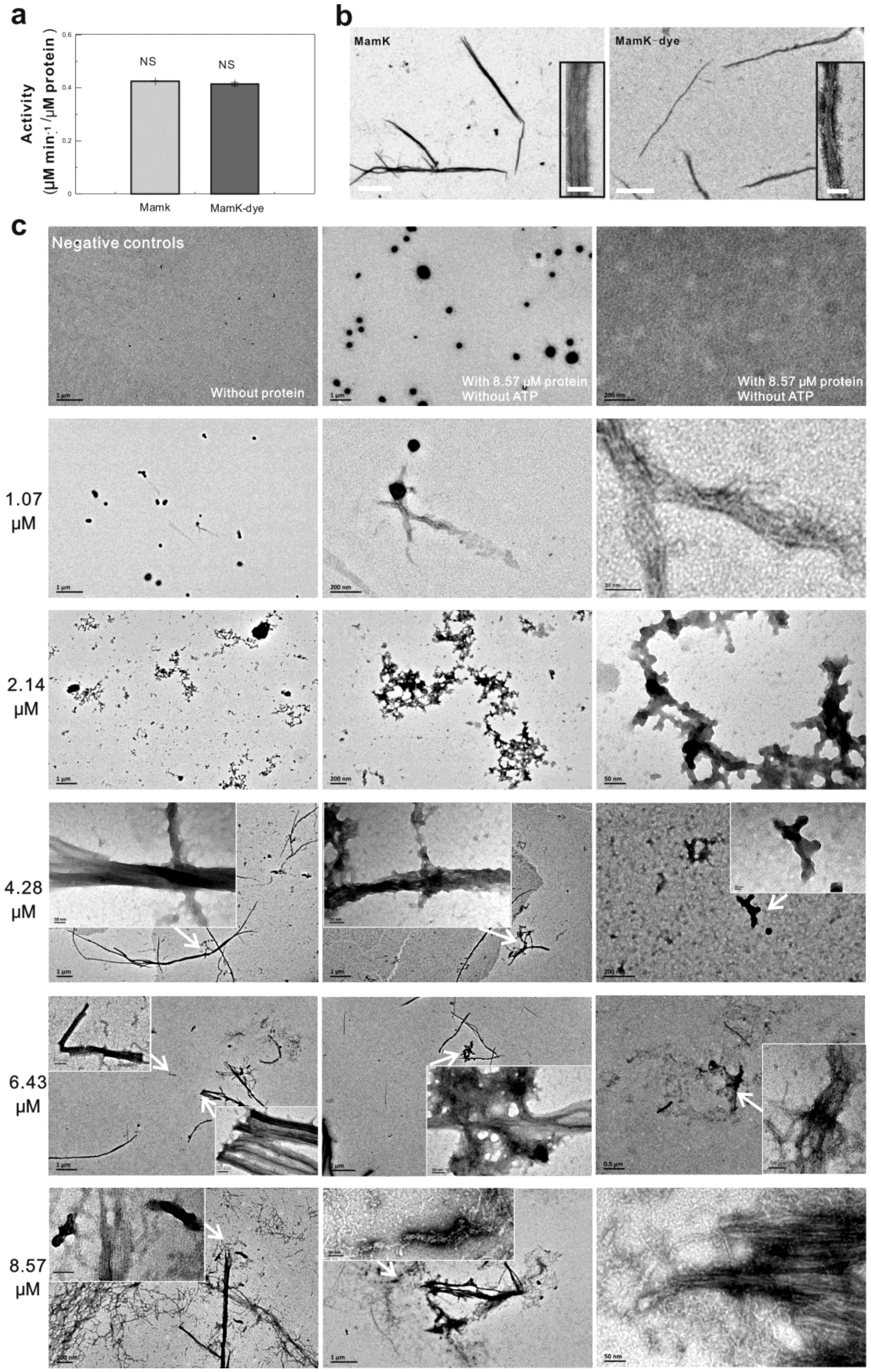

Supplementary Figure S6 | The ATPase activity and filament structure of Alexa 488-labeled proteins. (a) The ATPase activities of MamK and MamK-dye were

assayed based on phosphate release. MamK labelled with Alexa 488 displayed similar catalytic efficiency towards ATP as that of the unlabelled protein. Results are given as the means ± SD (n=3); NS, not significant (Duncan's multiple range test, $p<0.05$). (b) The filament structures formed by MamK and MamK-dye. The insets show high-magnification images. The 0.2 μm and 50 nm scale bars are shown in the low and high magnification images, respectively. (c) The filament structures formed by different concentration of MamK protein doped with 15% Alexa 488-labelled monomer. The polymerization experiments similar to that of filament polymerization kinetics were performed on TEM grids with a poly-L-lysine layer. No filament formation was observed in the absence of nucleotide or protein. Some small polymers were observed in the presence of 1.07 μM protein. Numbers of polymers were formed in the presence of 2.14 μM protein and many of them were interwoven or twisted. When the protein concentration increased to 4.28 to 6.43 μM, the well-developed filamentous bundles are observed and some of them were mixed with the irregular polymers. In the presence of 8.57 μM protein, numbers of filamentous bundles were observed.

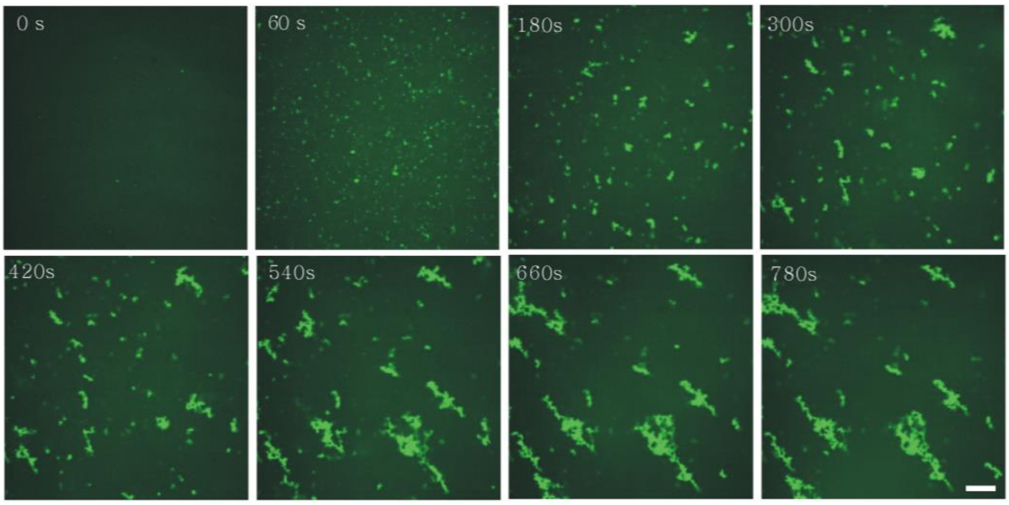

Supplementary Figure S7 | Polymerization process of the MamK filament assembly by fluorescence microscopy. The fluorescent intensity was increased after MamK was added to the reaction buffer, and large numbers of dotted polymers formed after 60 s. Then, these polymers started to elongate, and some of them formed long filaments between 60 and 420 s. After 420 s, the filaments interacted with each other and formed long, warped structures with multiple bundles. The scale bar is 2.5 μm.

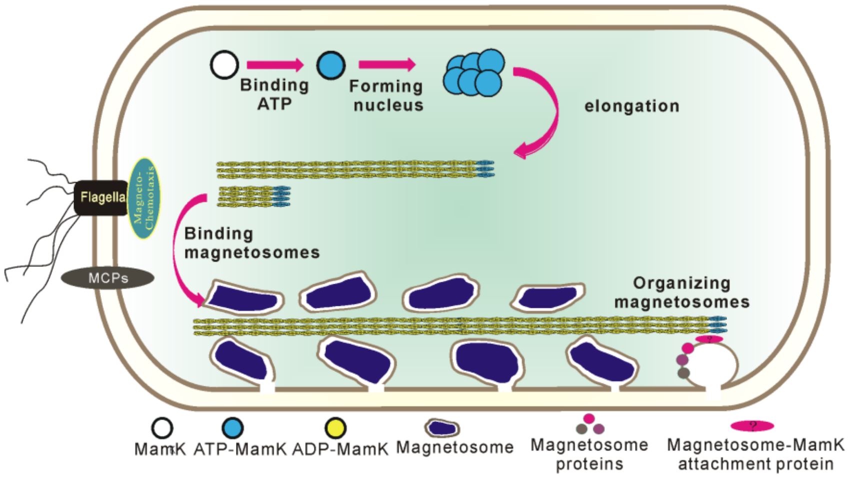

Supplementary Figure S8 | Model for dynamically stable assembly and the putative molecular behavior of MamK filaments. According to the MamK filament assembly behavior *in vitro*, the putative function of MamK in genus '*Ca.* magnetobacterium' has been speculated. First, MamK monomers might bind to the ATP and form ATP-MamK monomers once MamK molecules are synthesized. Then, the ATP-MamK molecules would nucleate spontaneously and might create trimeric nuclei. Subsequently, the nuclei could elongate and the filaments might form. Finally, the filaments would attach to the magnetosome membrane directly or through mediator protein(s), and the magnetosomes might be ultimately organized into multiple chains. Taken together, MamK monomers could bind ATP, might form a three-monomer nucleus and further elongate to form filaments.
