# Supplementary material for: In vitroassembly of the bacterial actin protein MamK from ‘CandidatusMagnetobacterium casensis’ in the phylumNitrospirae
Source: Protein Cell. 2016 Mar 9;7(4):267–80. doi: 10.1007/s13238-016-0253-x (PMC4818849; doi:10.1007/s13238-016-0253-x)
Supplement: Supplementary file 2 — Supplementary material 2 (PDF 378 kb) [file 13238_2016_253_MOESM2_ESM.pdf]

Supplementary Material for

In vitro assembly of the bacterial actin protein MamK from '*Candidatus* Magnetobacterium casensis' in the phylum *Nitrospirae*

Aihua Deng[1], Wei Lin[2], Nana Shi[1], Jie Wu[1,3], Zhaopeng Sun[1,3], Qinyun Sun[1,3], Hua Bai[1,3], Yongxin Pan[2], Tingyi Wen[1,*]

[1]CAS Key Laboratory of Pathogenic Microbiology and Immunology, Institute of Microbiology, Chinese Academy of Sciences, Beijing 100101, China

[2]Biogeomagnetism Group, Paleomagnetism and Geochronology Laboratory, Key Laboratory of Earth and Planetary Physics, Institute of Geology and Geophysics, Chinese Academy of Sciences, Beijing 100029, China

[3]University of Chinese Academy of Sciences, Beijing 100049, China

[*]Correspondence should be addressed to T. W. (wenty@im.ac.cn.)

Supplemental Methods

Cloning, expression and purification. The *mamK* gene was amplified from Mcas genomic DNA using the primers WB643 (AAGGCATATGACAAAAACAAAGATACTTAACA) and WB567 (TTCAAGCTTTTATCGCTTGCTTACTTCGTCCCA) and then digested with *Nde*I and *Hin*dIII. The digested products were purified and ligated with the pET28a vector (Invitrogen) and digested with the same restriction enzymes to create the plasmid pWYE369 for expressing N-terminal his-tagged MamK. To construct C-terminal his-tagged MamK expressing plasmid pWYE403, the *mamK* gene was amplified using primers WB643 and WB860 (GTGAAGCTTTCGCTTGCTTACTTCG) and then cloned into the pET22b vector (Invitrogen) at restriction sites *Nde*I/*Hin*dIII. The plasmids pWYE369 and pWYE403 were transformed into *Escherichia coli* C43 (DE3) cells and verified by DNA sequencing. The cells were grown in LB medium at 37 ℃ and induced with 0.5 mM IPTG after reaching an OD_{600} of 0.8. After 4 hours of induction, the cells were harvested and disrupted by sonication in buffer A containing 150 mM NaCl, 10% glycerol and 20 mM Tris-HCl (pH 8.0). After centrifugation at 15,000 ×*g* for 30 min, the his-tagged MamK protein in the supernatant was purified by Ni^{2+} affinity chromatography (GE healthcare, USA) and gel filtration (Superdex 75 10/300 GL high-performance column, GE healthcare, USA). To obtain protein without his tag, purified N-terminal his-tagged MamK was subsequently incubated with immobilizing bovine thrombin (Sigma, USA) at 4 ℃ to

remove the poly-histidine tags. The resulting MamK protein only with three residues at the N-terminus was eluted from the thrombin-agarose, and passed through a Ni^{2+} affinity column again to remove the remove uncleaved protein and the cleaved His tags. The purified protein was centrifuged at 500,000 ×*g* for 30 min at 4 ℃ to remove the aggregates and stored in aliquots at –80 ℃ for further study. The protein concentration was determined using the BCA protein assay kit (Pierce, Rockford) with bovine serum albumin (BSA) as the standard, and the sample purity was assessed by sodium dodecyl sulfate polyacrylamide gel electrophoresis (SDS-PAGE) (Supplementary Fig. S1).

The degree of labelling. The degree of labelling was determined after measuring the absorbance of the purified solution at 280 nm and 494 nm (A_{280} and A_{494} for detecting the concentrations of the protein and dye, respectively) using a Nanodrop 2000C spectrophotometer (Thermo Scientific, USA). The protein concentration (M) in the samples was calculated as equation 1:

$$M = \frac{\left[A_{280} - (A_{494} \times 0.11)\right] \times n}{25120} \qquad (1)$$

where n is the dilution factor and 25,120 $cm^{-1}\,M^{-1}$ is the molar extinction coefficient of the MamK protein at 280 nm (estimated using the ProtParam tool, http://web.expasy.org/protparam/). The degree of labeling (D, moles dye per mole protein) was calculated as equation 2:

$$D = \frac{A_{494} \times n}{71000 \times M} \qquad (2)$$

where n is the dilution factor and 71,000 cm^{-1} M^{-1} is the molar extinction coefficient of the Alexa Fluor 488 dye at 494 nm.

Characterization of MamK ATPase features. Enzyme activity was determined using a modified malachite-green assay as described above, with slight further modifications. Purified protein at a final concentration of 2.0 μM in the nucleotide hydrolysis reaction was used to determine ATPase activity under various conditions. The optimum temperature was determined after incubating the reaction mixtures at different temperatures (30-70 ℃). The effect of pH on enzyme activity was measured at different pH values (5-11) at 37 ℃. The relative enzyme activity was calculated as a percentage of the maximal activity. For the thermal stability assay, MamK was incubated at various temperatures (30-70 ℃) for 30 min, and the residual enzyme activity was subsequently determined. The residual enzyme activity was calculated as a percentage of the starting activity.

The effects of various metal ions on the enzyme activity were determined after incubating the reaction mixtures with $MgCl_2$, $CaCl_2$, $FeSO_4$, $CuCl_2$ and $NiSO_4$ at concentrations ranging from 0 to 2.0 mM. To avoid the oxidation of iron (II), $FeSO_4$ was dissolved in the reaction buffer containing a reducing agent (2 mM ascorbic acid), which had been determined to have no effects on the MamK ATPase activity. The enzyme activity assayed under the same condition, but in the absence of metal ions, was set at 100%.

To evaluate the effect of salt, the enzyme activity was measured in the presence of 0-500 mM KCl and NaCl. The enzyme activity assayed under the same conditions, but in the absence of salts, was set at 100%.

The kinetic parameters were determined through Lineweaver-Burk curves using varying concentrations of ATP from 0.01 to 0.50 mM. Substrates at different concentrations were incubated with MamK using a standard ATPase activity assay as described above. The velocity (μM min^{-1} Pi /μM protein, y axis) was defined as the change in catalytic rate of the concentration of released phosphate per minute and per μM protein, which was plotted against the concentration of the substrate (μM, x axis). Subsequently, the curves were fitted using the Lineweaver-Burk curve method, from which the Michaelis constants (*Vmax* and *Km*) for each enzymatic reaction were derived. The initial velocities were used to determine *Vmax* and *Km*. The catalytic constant (*Kcat*) was determined after dividing the *Vmax* by the concentration of the enzyme. The catalytic efficiency was defined as *Kcat*/*Km* (min^{-1} μM^{-1}).

Determination of the physicochemical conditions for polymerization. Various parameters affecting MamK assembly were determined in the polymerization reaction mixture (20 mM Tris-HCl (pH 8.0), 1 mM $MgCl_2$ and 50 mM NaCl) with slight modifications. Based on the biochemical properties of the MamK ATPase activity, the essential parameters (nucleotide substrates, temperatures ranging from

20 to 50 ℃, divalent cations and varying salt concentrations) were used to characterize the assembly properties of MamK. ATP, GTP, ATP-γ-S (adenosine-5′-(γ-thio) triphosphate) and AMP-PNP (adenosine 5′-(β, γ-imido) triphosphate) were used to determine the optimal nucleotide substrate for MamK assembly. To evaluate the effect of salts, MamK filaments were assembled in the presence of 0-200 mM of KCl and NaCl. Polymers generated under the same conditions in the absence of salt were used as controls. To assess protein aggregation during the reaction, a negative control without nucleotides was assayed under the same conditions as that of the polymerization reaction, except for the presence of the nucleotide.

Fluorescence microscopy. To image the protein assembly, MamK was conjugated to the fluorescent probe Alexa 488 as described above. The cover slips were treated with 0.1% poly-L-lysine for 2 hours and washed with 0.3 ml of ddH_2O. Approximately 4.5 μM of MamK was doped with 15% Alexa 488-labelled monomer in polymerization buffer and loaded onto a cover slip, followed by polymerization using saturating amounts of ATP at 37 ℃. The fluorescently labelled filaments were observed at 0 to 25 μm from the bottom of the slide using a Leica SP8 confocal microscopy.

Determination of the polymerization by pelleting assay. Because the ATPase

activity and assembly of MamK from Mcas was inhibited at 4 ℃ (Fig. 3a), polymerization was assessed using a pelleting assay. To simultaneously determinate the ATP hydrolysis and the polymerization of MamK protein, the polymerizing sample at the same time point was separated in two parts. One was mixed with 4 volumes of chromogenic reagent and incubated at 37 ℃ for 30 min, while another was centrifuged at 500,000 ×*g* at 4 ℃ for 30 min. Subsequently, they were subjected to determine the amount of Pi (630 nm) and protein monomers concentration (595 nm) by spectrophotometer and the pellet by SDS-PAGE.

Statistical analyses. Significant differences were analyzed using SPSS software (version 13.0; http://www-01.ibm.com/software/analytics/spss/). The data conducted under various conditions were compared by one-way analysis of variance (ANOVA) and Duncan's multiple range tests. All measurements were performed at least in triplicate, and each value represented the mean ± standard deviation (SD). P-values less than 0.05 were considered statistically significant.
