# Supplementary material for: In vitroassembly of the bacterial actin protein MamK from ‘CandidatusMagnetobacterium casensis’ in the phylumNitrospirae
Source: Protein Cell. 2016 Mar 9;7(4):267–80. doi: 10.1007/s13238-016-0253-x (PMC4818849; doi:10.1007/s13238-016-0253-x)
Supplement: Supplementary file 3 — Supplementary material 3 (PDF 128 kb) [file 13238_2016_253_MOESM3_ESM.pdf]

Supplementary Table S1 | Proteins used for constructing phylogenetic tree.

Proteins	The sources of organism	Accession numbers (GenBank)
MamK	*Magnetospirillum magneticum* AMB-1	83310064
	M. gryphiswaldense MSR-1	568146778
	M. magnetotacticum RS-1	ZP_00054405.2 (NCBI RefSeq)
	Magnetovibrio blakemorei MV-1	238653874
	Gamma proteobacterium SS-5	425483541
	Desulfovibrio magneticus RS-1	239908729
	Candidatus Desulfamplus magnetomortis BW-1	356601823
	Delta proteobacterium ML-1	429143452
	Ca. Magnetoglobus multicellularis	317383429
	Magnetospira sp. QH-2	578036001
	Magnetococcus marinus MC-1	117925549
MreB	*Thermotoga maritima*	15988309
	T. maritima	15643354
	Escherichia coli K-12	388479242
	E. coli	486290201
	Bacillus subtilis	142855
	M. magneticum AMB-1	83312612

	Caulobacter crescentus	16125790
ParM	*Klebsiella oxytoca*	556470232
	Serratia marcescens	671769802
	B. thuringiensis T01-328	542047572
	B. cereus ATCC 10987	42738147
	E. coli	410609677
	E. coli (ParM-R1)	134954
	E. coli (ParM-pB171)	10955418
	Salmonella enterica (ParM-R64)	32470180
	S. enterica (ParM-R621a)	345134017
Alp12	*Clostridium tetani*	28373143
AlfA	*B. subtilis*	323651003
Alp7A	*B. subtilis*	323651170
FtsA	*T. maritima*	499163367
	C. crescentus CB15	16126780
	E. coli	190905138
	M. magneticum AMB-1	83312952
	B. subtilis	255767352
Actin	*Saccharomyces cerevisiae* S288c	14318479
	Homo sapiens	4501881
	Oryctolagus cuniculus	411024247

H. sapiens	4501885
Caenorhabditis elegans	17568985
Drosophila melanogaster	17530805
Giardia lamblia ATCC 50803	159108769
